# Chloral Hydrate Alters Brain Activation Induced by Methamphetamine-Associated Cue and Prevents Relapse

**DOI:** 10.3389/fnmol.2022.934167

**Published:** 2022-07-11

**Authors:** Chenyu Jiang, Yunlong Xu, Jiafeng Zhong, Junyan Wu, Jian He, Wei Xu, Yingjie Zhu

**Affiliations:** ^1^Shenzhen Key Laboratory of Drug Addiction, Shenzhen Neher Neural Plasticity Laboratory, The Brain Cognition and Brain Disease Institute, Shenzhen Institute of Advanced Technology, Shenzhen-Hong Kong Institute of Brain Science-Shenzhen Fundamental Research Institutions, Chinese Academy of Sciences, Shenzhen, China; ^2^College of Life Science, University of Chinese Academy of Sciences, Beijing, China; ^3^The First Affiliated Hospital, Sun Yat-sen University, Guangzhou, China; ^4^Medical College of Acupuncture-Moxibustion and Rehabilitation, Guangzhou University of Chinese Medicine, Guangzhou, China; ^5^Department of Anesthesiology, The First People’s Hospital of Foshan, Foshan, China; ^6^Faculty of Life and Health Sciences, Shenzhen Institute of Advanced Technology, Chinese Academy of Sciences, Shenzhen, China; ^7^CAS Center for Excellence in Brain Science and Intelligence Technology, Chinese Academy of Sciences, Shanghai, China; ^8^CAS Key Laboratory of Brain Connectome and Manipulation, Brain Cognition and Brain Disease Institute (BCBDI), Shenzhen Institute of Advanced Technology (SIAT), Chinese Academy of Sciences, Shenzhen, China

**Keywords:** chloral hydrate (CH), methamphetamine, brain activation, cue-induced relapse, addiction

## Abstract

Methamphetamine is a highly addictive drug and its abuse leads to serious health and social problems. Until now, no effective medications are yet available for the treatment of methamphetamine addiction. Our study reveals that chloral hydrate, a clinical sedative drug, suppresses the seeking desire for methamphetamine. After 5 days of continuous administration (subanesthetic dose 50 mg/kg and 100 mg/kg), methamphetamine-seeking behavior of rats was inhibited in the condition place preference and intravenous self-administration tests. Furthermore, chloral hydrate treatment robustly suppressed cue-induced methamphetamine relapse. The whole brain c-fos immunostaining revealed that chloral hydrate treatment suppressed neuronal activity in the rhomboid thalamic nucleus (Rh), dorsal endopiriform nucleus (dEn), and claustrum (Cl) while enhanced zona incerta (ZI) activity during cue-induced methamphetamine relapse. Therefore, chloral hydrate could remodel neural network activity and serve as a potential medicine to treat methamphetamine addiction.

## Introduction

Drug addiction is a chronic brain disease that is characterized by compulsive drug-seeking and unavoidable relapse. Relapse would be triggered by drugs, stress, and cues even after long-term abstinence. The changes in neuroplasticity are the biological basis of drug relapse. Multiple neurocircuits involve in the development of drug relapse. Thus, it is important to identify the activation of the neural network in drug relapse and develop therapeutic targets.

Methamphetamine (Meth) abuse is gradually increasing worldwide (Ballester et al., 2017). Chronic methamphetamine abuse can lead to malnutrition, psychosis, mania, aggression, and other medical and psychiatric symptoms such as cardiovascular complications, especially during withdrawal (Scott et al., 2007). The addictive properties of Meth are believed to be mediated by elevating the synaptic cleft dopamine concentration in the mesolimbic area (Sulzer et al., 2005). Although several behavioral therapies have been used to treat Meth use disorders, the effects are limited in clinics (Ballester et al., 2017). There are no FDA-approved drug treatments for Meth addiction to date, and clinical trials have not identified compounds with clear efficacy. Thus, it is urgent to develop effective drugs to treat Meth addiction.

Chloral hydrate (CH) is one of the most commonly used sedatives for non-invasive procedure in clinic. It slows the activity of the whole central nervous system without a clear mechanism. Although CH has been used as a sedative for decades, it is almost unknown about its other applications. CH is able to enhance GABAa receptor function, thus it is potential to modulate the progression of drug addiction (Lu et al., 2008). In one retrospective study (Esmaeili et al., 2010), chloral hydrate was co-administered with clonidine to ameliorate opiate withdrawal symptoms and neonatal narcotic abstinence syndrome in neonates delivered by addicted mothers. In morphine-dependent rats, preceding treatment with a single injection of chloral hydrate decreased the withdrawal symptoms induced by naloxone (Streel et al., 2000). At the anesthetic dose, CH was potential to inhibit dopamine transporter function in the dorsal striatum as measured by high-speed chronoamperometry (Sabeti et al., 2003). In another study, CH did not affect the basal and cocaine-induced increases of dopamine levels, but decreased glutamatergic transmission in the striatum (Kreuter et al., 2004). In addition, the active metabolite of chloral hydrate-trichloroethanol (TCE) was able to increase the firing frequency of dopamine neurons in the ventral tegmental area (VTA) (Appel et al., 2006).

Considering the effects on dopamine system, we try to explore that the role of CH plays in Meth addiction. We comprehensively employed the conditioned place preference paradigm and self-administration paradigm to evaluate its role in drug addiction. We further observed the alteration in neural network activity by CH during cue-induced Meth reinstatement.

## Materials and Methods

### Animals

Male Sprague–Dawley (SD) rats were purchased from Charles River (Beijing, China) at 6 weeks of age. Rats were pair-housed in a constant temperature (22 ± 1) and humidity-controlled (45 ± 5%) environment *ad libitum*. Lighting was maintained under a 12-h light–dark cycle (lights on from 8:00 am to 8:00 pm). The rats were habituated at least 14 days prior to conditioned place preference (CPP) and Meth self-administration (SA) experiments. The experimental procedures were performed in accordance with the guidelines for the National Care and Use of Animals, and the experiments were approved by the Institutional Animal Care and Use Committee (IACUC) of Shenzhen Institute of Advanced Technology. All efforts were made to minimize animal suffering.

### Drugs

Chloral hydrate (Sigma, Shanghai, China) was dissolved in sterile saline as 100 and 200 mg/ml. Methamphetamine hydrochloride supplied by the National Institutes for Food and Drug Control/NIFDC (171212-201605) was dissolved in sterile saline as 0.25 mg/ml solution. The volume of intraperitoneal injection at 2.0 ml/kg.

### Apparatus (CPP)

For place conditioning (a total of 24 male SD rats were used), we employed four identical polypropylene (PE) boxes with two equal-sized compartments (30 cm × 25 cm × 30 cm) separated by a black central area (10 cm × 25 cm × 30 cm). The compartments had different pattern walls (black and white column vs. black and white square) and distinct floor textures (fine grid in the column compartment and sandpaper in the square one). An industrial wide-angle camera was used to record the experiment video (LRCP, trade ID:3206). Anymaze software was adopted to analyze videos, and Deeplabcut software (from Harvard University) was used to trace the animal exercise trajectory.

### Apparatus (Meth SA)

Operant chambers (AES-DSA6, Anilab) situated in sound-attenuating boxes (Anilab) were used for operant tasks. Self-administration chambers were equipped with two illuminated nose pokes (“active” and “inactive”), a house light, and infusion pump. The two nose poke operands were set on the front wall of the chamber. The stimulus light and sound cue were positioned directly above the active nose poke hole. The house light was set on the rear wall of the chamber and, except for 20 s timeout periods, the house light remained on when rats were inside the operant chamber. The apparatus was controlled and data were captured by Anilab Ver 6.40 software (Anilab).

### Apparatus (OFT)

The open field test (a total of 15 male SD rats were used) was comprised of two identical PE equal-sized boxes (50 cm × 50 cm × 50 cm). The central area was defined as a 30 cm × 30 cm square. An industrial wide-angle camera (LRCP, trade ID:3206) was used for recording. The Anymaze software was used to analyze the video data, and Deeplabcut software (from Harvard University) was used to trace the trajectory.

### Intravenous Catheterization

Rats were anesthetized with isoflurane (RWD; cat. no. R510-22-10). A silicon catheter (Dow, cat. no. 508-003) with rounded tip and double suture beads (one secured internally and other externally) was implanted into the right external jugular vein. The distal end of the catheter was subcutaneously placed around the shoulder and exited below the scapula *via* polycarbonate back-mount access port (PlasticsOne; C313G-5UP). Immediately after the surgery, catheters were flushed with 0.2 ml of gentamicin sulfate (2 mg/ml; Sigma; cat. no. E003632) diluted in sterile heparinized saline (10 U/ml; Tocris; cat. no. 2812/100). Atipamezole hydrochloride (0.5 mg/kg; IM; Sigma), diluted in saline was used to terminate anesthesia. Rats were given 5 days to recover from surgery before starting the experiment. During recovery, rats had *ad libitum* access to food for the first 4 days and then gradually food restricted to 90%. Catheters were daily flushed with heparinized saline (10 U/ml). Catheter patency was assessed with a 0.05 ml IV infusion of xylazine (20 mg/ml) at the end of methamphetamine self-administration phase or when patency loss was suspected. Only rats with patent catheters were adopted in the following experiments.

### Conditioned Place Preference

Place conditioning consisted of three phases. In the first phase (or preconditioning, briefly named Pre-C), rats were allowed access to both compartments of the apparatus for 900 s per day on the first day. On day 2, the time spent in each compartment was recorded (test 0). The chamber in which mice spent less time will be selected as drug-paired compartment.

In the second phase (conditioning), which lasted 8 days, saline was paired with the preferred compartment and Meth was paired with the other compartment. Rats received 0.5 mg/kg Meth (*i.p.*) and saline injection alternately during this phase. Immediately after the injection, rats were restricted to the appropriate compartment with closed guillotine door for 45 min.

In the third phase (or postconditioning, briefly named Post-C, day 11, test 1), the CPP was measured. The CPP score was calculated as the time spent in the drug-paired compartment during the Post-C session minus the time spent in this compartment during the Pre-C session.

After the acquisition of CPP, rats received chloral hydrate (50 and 100 mg/kg) or saline (similar volume to chloral hydrate solution) injection (*i.p.*) during the maintenance stage. At this stage, rats stayed at home cage and received CH injection for 5 consecutive days. Then 24 h after the last injection, CPP was measured (test 2) to observe the effects of CH on the maintenance of CPP.

### Self-Administration

The rats were subjected to the protocol after recovery from the surgery.

### Acquisition

Experiments were conducted as previously described with slight modifications (Wakabayashi et al., 2010). The rats were trained for 12 daily fixed ratio 1 sessions (2 h/day). During the acquisition sessions, active nose pokes (termed fixed-ratio one, or FR1) triggered the infusion pump (0.2 mg/kg/infusion), 5-s continuous voice from the buzzer and the illuminated the nose poke. A 20-s time-out was signaled by the dimming of the house light. We set 50 infusions as the maximum per session to avoid drug overdoses. The rats with stable infusion (less than 20% variance over the final 3 days of self-administration training) were selected for subsequent experiments.

### Maintenance

In the maintenance phase, the rats were randomly divided into two groups: saline group and CH group. The rats continued daily self-administration training, and received chloral hydrate (100 mg/kg, 2 ml/kg) or saline (2 ml/kg) injection (*i.p.*) immediately after they finished daily training session of 5 consecutive days (day 16–20). Then, we tested the effects of CH on the Meth self-administration on days 21, 22, 23, 27, 34, and 42.

### Extinction and Cue-Induced Reinstatement

The experiment is designed according to the previous literatures with slightly modifications (Allain et al., 2021; Everett et al., 2021). During extinction, rats underwent 60 min training sessions, in which nose poke resulted in no scheduled consequences, but poke data were recorded. After the first extinction session, rats were subjected to the maintenance injection protocol. The extinction phase comprised 10 consecutive sessions. After the extinction phase, rats with active poke no more than 10 for 3 consecutive sessions were chosen for the reinstatement test.

During the reinstatement phase, a conditional cue (same as the acquisition phase) was given at the test beginning with 10 s. The active poke (termed fixed-ratio one, or FR1) resulted in the delivery of the same cue as the acquisition phase except no drug infusion. Reinstatement sessions lasted 1 h.

### Treatment of Chloral Hydrate

Chloral hydrate was dissolved in sterile saline. In the rat’s Meth CPP experiments, after CPP was established, chloral hydrate was given daily at doses of 50 or 100 mg/kg for continuous 5 days. In the rat’s Meth self-administration experiments, chloral hydrate was given (*i.p.*) daily at doses of 100 mg/kg immediately after the training sessions for continuous 5 days. In the SA extinction experiments, chloral hydrate was given (*i.p.*) daily at doses of 100 mg/kg immediately after the extinction training sessions for continuous 5 days.

### Sucrose Self-Administration

The rats were trained for 12 daily fixed ratio 1 sessions (2 h/day). During the acquisition sessions, active nose pokes (termed fixed-ratio one, or FR1) triggered the infusion pump (0.04 mg/infusion, 200 μl/infusion), 5-s continuous voice from the buzzer and the illuminated the nose poke. A 20-s time-out was signaled by the dimming of the house light. We set 100 infusions as the maximum per session. The rats with stable infusion (less than 20% variance over the final 3 days of self-administration training) were selected for subsequent CH or saline treatments.

### Open Field Test

After the sucrose self-administration test, rats received 100 mg/kg chloral hydrate treatment for continuous 5 days. The open-field test was conducted 24 h after the last chloral hydrate injection. The rats were put in the OFT chamber central area and explored freely for 15 min. The traveling distance and staying in central time will be recorded and analyzed by Anymaze software.

### c-fos Immunostaining

Then, 1 h after the cue-induced reinstatement test, rats were deeply anesthetized by pentobarbital sodium (80 mg/kg, *i.p.*, Merck, cat. no. P11011) and perfused with phosphate buffered saline followed by 4% paraformaldehyde (PFA). Brains were postfixed overnight in 4% PFA and were sliced on a microtome at 45 μm. The slices were washed with 3 times TBST for 10 min and blocked with 5% goat serum dissolved in 0.3% TritonX-100 for 2 h at room temperature. Slices were incubated with primary antibodies dissolved in blocking buffer overnight at 4°C, followed by 3 times 10 min wash with TBST at room temperature. After that, slices were incubated with fluorescent-conjugated secondary antibodies dissolved in blocking buffer for 2 h at room temperature and washed 3 times with TBST. Then, slices were incubated with DAPI in PBS for 10 min at room temperature followed by 3 times 10-min wash with PBS. Finally, slices were mounted using mounting medium containing DAPI (Fluoroshiel, sigma). Primary and secondary antibodies are listed below:

Rabbit anti c-fos (1:2,000, Synaptic Systems Cat# 226 008, RRID:AB_2891278); goat anti-Rabbit IgG 647 (1:1,000, Thermo Fisher Scientific Cat# A-21245, RRID:AB_2535813). Data analysis used the OLYMPUS cellSens Dimension (Ver 3.2, Build 23706). The c-fos immunostaining of each brain region was averaged by the c-fos of two adjacent brain slices and the Student’s *t*-test was used for statistical analysis.

### Clustering Analysis

Clustering analysis was performed with MATLAB (Mathworks). Prior to the clustering, the variables during the maintenance phase (baseline to day 42) of each rat were normalized to the average time during the baseline sessions (days 13–15). The variables used from the Meth self-administration were the number of active pokes, useless pokes, inactive pokes, and infusions. Useless pokes were defined as the poke to active pokes during the 20-s time-out period after Meth infusions. A dimension reduction (MATLAB toolbox UAMP with option n_neighbors = 10, min_dist = 0.5, metric = euclidean, n_components = 3) was applied to the variables followed by a hierarchical clustering method (MATLAB functions “pdist,” “linkage,” and “cluster” with a metric = s-euclidean and linkage = ward). Since the UMAP is a stochastic method, the dimension reduction and clustering were run 500 times and choose the best result to hierarchical clustering. Finally, other relevant variables were sorted according to the obtained clustering (treatment).

### Statistics

One-way ANOVA was applied to evaluate the effects of chloral hydrate dose (0, 50, and 100 mg/kg) using SPSS (version 25, General Linear Model Procedure). Student’s *t*-test was used to determine the effects on maintenance and relapse across CH treatment and after treatment in self-administration, and condition preference across CPP test using GraphPad Prism 8.4.7. Clustering analysis was conducted by MATLAB 2020b (MathWorks) for Windows. The minimum level of significance was set at *p* < 0.05. Data are presented as the mean ± SEM.

## Results

### Chloral Hydrate Treatment Attenuates the Maintenance of Methamphetamine-Induced Conditioned Place Preference

**FIGURE 1:**
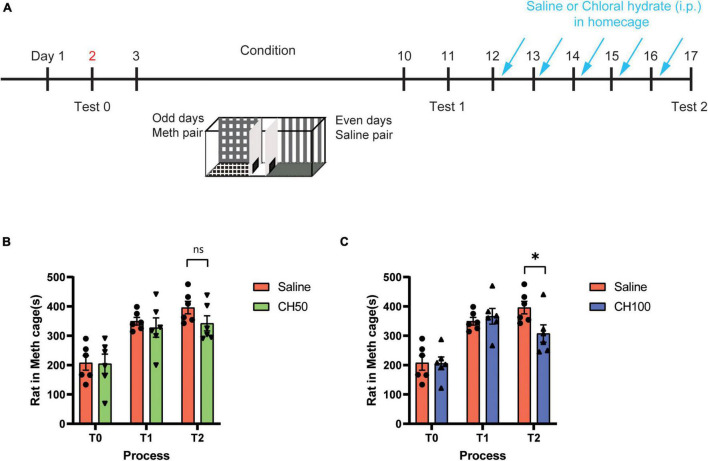
Chloral hydrate reduces Meth-induced CPP maintenance. **(A)** Representation of rat CPP schedule. Three-chamber system was adopted. **(B)** The effect of 50 mg/kg CH treatment (*N* = 6) on the maintenance of Meth-associated CPP memory. **(C)** The effect of 100 mg/kg CH treatment (*N* = 6) on the maintenance of Meth-associated CPP memory. *p < 0.05, ns: no significant difference. T0: test 0, T1: test 1, T2: test 2.

The conditioned place preference **paradigm** is a standard preclinical behavioral model used to evaluate the rewarding effects of the drugs. We first employed CPP paradigm to assess the impact of CH treatment on the rewarding property of Meth (Figure 1A). Rats were habituated to 3-chambers CPP apparatus on day 1, and the baseline was assessed on day 2 (test 0). After 8 days of alternating saline and Meth pairing, a CPP expression test (test 1) was performed on day 11 to evaluate the Meth-associated memory. Then, rats were subjected to successive 5 days of saline or chloral hydrate treatment (50 and 100 mg/kg, *i.p.*). A second CPP test was performed to examine the impact of CH treatment on the CPP maintenance (test 2).

After 8 days CPP training, rats increased their time spent on Meth-paired side on test 1, suggesting the formation of Meth-associated CPP [F_(1, 10)_ = 26.58, *p* < 0.001, Figures 1B,C]. After 5 days of CH injection, the 50 mg/kg CH treatment group showed slight decrease in the time spent on Meth-paired side compared to saline-treated group, but did not reach significant level (*p* = 0.14, Figure 1B). However, 100 mg/kg CH treatment significantly decreased the time spent on Meth-paired side (*p* < 0.05, Figure 1C). These results indicated that 100 mg/kg of CH treatment for 5 days is sufficient to attenuate Meth-associated CPP memories. Based on these results, we chose to use a dose of 100 mg/kg for subsequent studies.

### Chloral Hydrate Treatment Reduces Methamphetamine-Seeking Behavior in Drug Self-Administration Model

**FIGURE 2:**
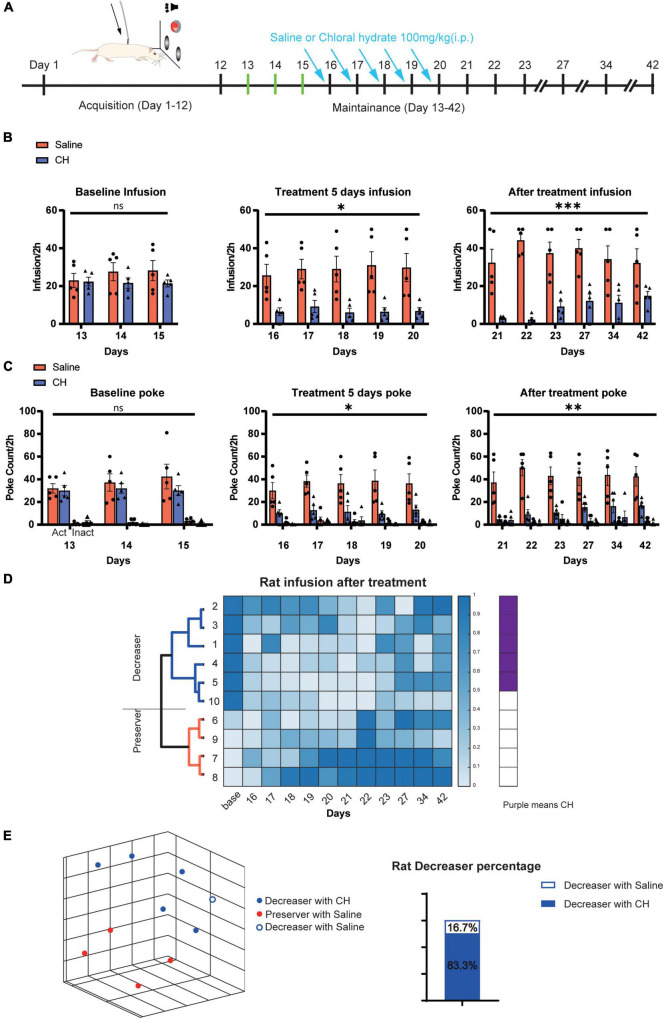
Chloral hydrate reduces drug-seeking behavior in Meth self-administration. **(A)** Representation of rat self-administration schedule. **(B,C)** Chloral hydrate reduced drug-seeking behaviors (*N* = 5 in the CH100 group, *N* = 5 in the saline group). **(D)** Hierarchical cluster based on uniform manifold approximation and projection (UMAP) of different parameters of maintenance session of Meth SA. Purple means CH100 treatment rats. The dark and light blue colors represent the normalized number of the infusion count. **(E)** The representation of three-dimensional UMAP clusters of decreaser (cluster blue) and preserver (cluster red) after treatment in Meth SA. *p < 0.05, ^**^p < 0.01, ^***^p < 0.001; two-way ANOVA.

We next examined the effect of chloral hydrate treatment on the Meth-seeking behavior with classical intravenous drug self-administration (SA) paradigm (Figure 2A). Then 7 days after jugular vein cannulation surgery, rats were subjected to 12 days of SA acquisition training. Rats that met the requirements of the successful self-administration were selected and randomly assigned to saline and CH groups. After 12 days of training, the saline group and CH group showed similar level of nose poking and Meth infusion (Figures 2B,C, left). During the maintenance phase, rats were treated with saline or CH (100 mg/kg) immediately after SA session and 22 h before the next SA session for successive 5 days. CH treatment reduced the nose pokes and the drug infusions [Infusion count: F_(1, 8)_ = 11.09, *p* < 0.05, active poke count: F_(1, 8)_ = 10.03, *p* < 0.05, Figures 2B,C, middle].

To examine how long the effect could last after the cessation of CH treatment, the rats were returned to apparatus to perform SA task 1 day, 2 days, 3 days, 1 week, 2 weeks, and 3 weeks after the last CH treatment. The Meth infusion and nose pokes in CH treatment group remained low [infusion count: F_(1, 8)_ = 26.35, *p* < 0.001, active poke count: F_(1, 8)_ = 15.02, *p* < 0.01, Figures 2B,C right], and the effect was still significant 3 weeks after the last CH treatment.

Parallel to the statistical analysis of *a priori* labeling of animal groups, a dimensionality reduction hierarchical clustering analysis based on the UMAP algorithm was performed. An unbiased clustering analysis integrating four self-administration behavioral parameters over the chloral hydrate delivery sessions yielded two clusters: decreasers (means drug-seeking behavior reduces) and perseveres (means drug-seeking behavior remains unchanged or increases, refer to Figures 2D,E). Out of 6 decreaser rats, 5 (83.3%) were classified into the CH100 treatment group. Correspondingly, 4 of the 5 rats in the control group were the perseveres. These results indicated that chloral hydrate had a positive effect on the control of methamphetamine drug-seeking behavior during the maintenance phase and the effect was kept for a long time after the cessation of chloral hydrate.

### Chloral Hydrate Treatment Prevents Cue-Induced Relapse Without Affecting the Extinction of Methamphetamine-Seeking Behavior

**FIGURE 3:**
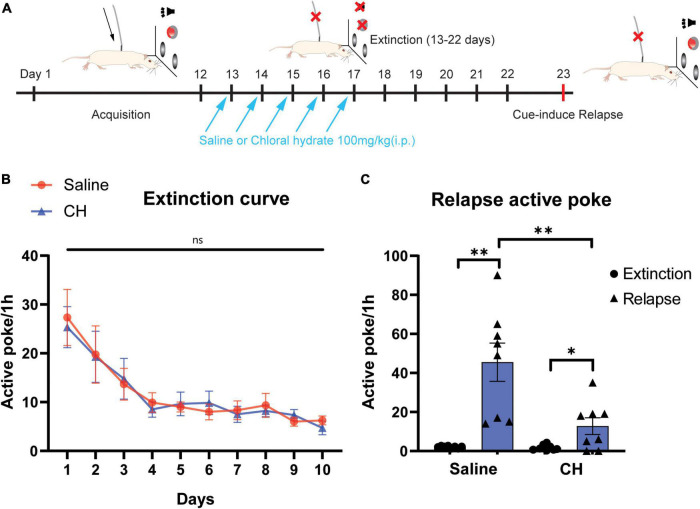
Chloral hydrate reduced cue-induced relapse without affecting extinction in Meth self-administration model **(A)** Representation of extinction and relapse schedule. **(B)** Chloral hydrate treatment (*N* = 12) has no effect on drug extinction, compared with saline control (*N* = 12). **(C)** Chloral hydrate treatment (*N* = 8) prevents cue-induced Meth relapse (*N* = 8 for CH group and N = 8 for saline group). *p < 0.05, ^**^p < 0.01, ns: no significant difference, Student’s *t*-test.

We further explored the effect of CH on the relapse of Meth-seeking behavior. We subjected animals to extinction training after they acquired stable Meth self-administration (Figure 3A). Rats were given either saline or CH treatment (100 mg/kg, *i.p.*) immediately after extinction training sessions for successive 5 days. After 10 days of extinction training (at this stage, nose poke does not trigger drug injection), the rats met that the extinction criteria would accept the cue-induced relapse test. Rats gradually decreased their nose pokes and reach successful extinction criteria (< 10 active pokes/hour) after 10 days of extinction training in the saline-treated group. At this stage, rats in the CH-treated group were indistinguishable in nose pokes from that of saline group [F_(1, 24)_ = 0.0085,*p* = 0.93, Figure 3B], indicating that CH is ineffective in facilitating drug extinction.

Then, the rats underwent cue-induced relapse test. The presentation of Meth-associated sensory cues induced robust active nose pokes in the saline control group, indicating successful reinstatement of Meth-seeking behavior (*p* < 0.01, Figure 3C). The CH-treated rats exhibited a significant lower number of nose pokes, suggesting CH treatment during extinction phase prevented cue-induced methamphetamine relapse [F_(1, 14)_ = 9.391, *p* < 0.01, Figure 3C].

### Chloral Hydrate Treatment Altered Whole Brain Neural Activation Induced by Methamphetamine-Associated Cue

**FIGURE 4:**
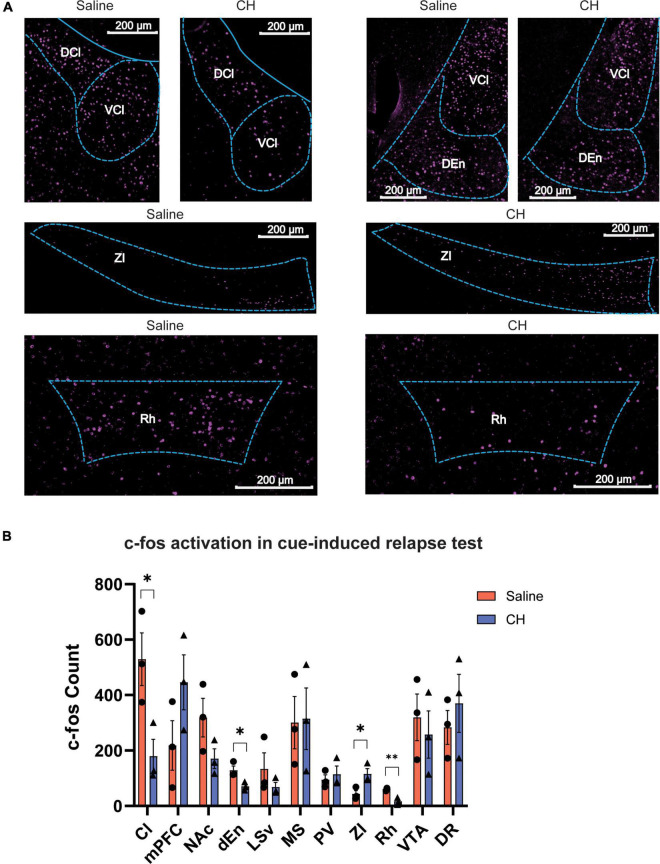
C-fos brain activation after cue-induced Meth relapse in saline- and CH-treated groups. **(A)** Example images showing c-fos expression of multiple brain regions after relapse test (*N* = 3 for both groups). **(B)** Quantification of brain regions with significant differences in c-fos expression between saline (red) and CH (blue) rats. *p*-values are presented in Supplementary Table 1. Abbreviations: rhomboid thalamic nucleus (Rh), dorsal endopiriform nucleus (dEn), zona incerta (ZI), claustrum (Cl), medial prefrontal cortex (mPFC), nucleus accumbens (NAc), lateral septal nucleus, ventral part (LSv), dorsal raphe (DR), medial septal nucleus (MS), paraventricular thalamic nucleus (PV), and ventral tegmental area (VTA). Student’s *t*-tests. *p < 0.05, ^**^p < 0.01.

The robust effect of CH on preventing cue-induced Meth relapse leads us to hypothesize that brain activation following cue presentation might be different between CH-treated and saline-treated group. We thus performed immunostaining of c-fos, a proxy of neural activity, to assess whole-brain activation induced by Meth-associated cues during relapse test. In the saline control group, cue-induced Meth relapse resulted in robust c-fos activation in many brain regions, including some brain regions that have previously been reported to be important for drug addiction, such as the medial prefrontal cortex (PFC), nucleus accumbens (NAc), and VTA (Figure 4A). Meth-treated animals showed some overlapping but distinct brain activation patterns (Figure 4A). By carefully comparing brain activation between the two groups of animals, we identified 4 brain regions with significant difference (Figure 4B). Among them, claustrum (Cl), dorsal endopiriform nucleus (dEn), and rhomboid thalamic nucleus (Rh) showed significant reduction in c-fos numbers in CH-treated animals, whereas zona incerta (ZI) showed significant increase in c-fos activation. In addition, PFC showed enhanced c-fos activation and NAc showed reduced activation in the CH group, but both did not reach significant level. These changes in brain activation might contribute to the suppression effect of CH on cue-induced Meth relapse.

### The Impact of Chloral Hydrate Treatment on Sucrose-Seeking, Locomotion, and Anxiety

**FIGURE 5:**
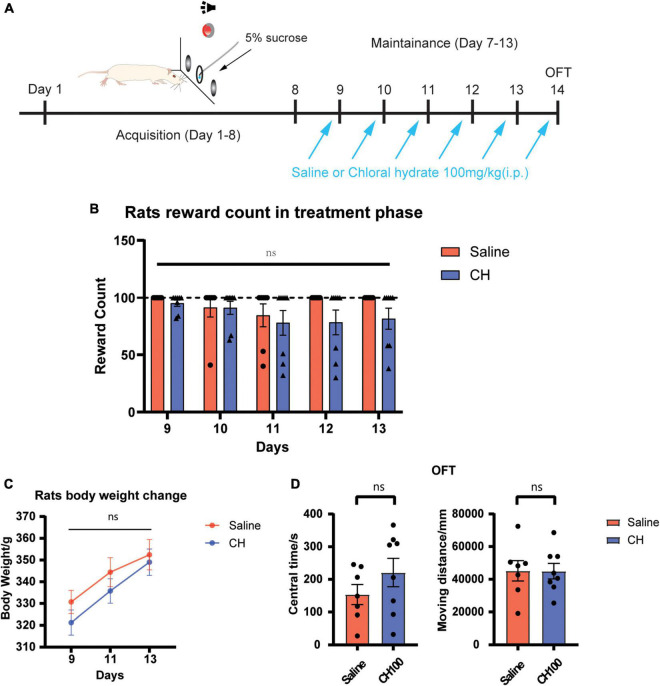
Chloral hydrate had no obvious side effects (*N* = 7 in Saline group and N = 8 in CH100 groups). **(A)** Timeline for sucrose self-administration. **(B)** Chloral hydrate did not affect sucrose self-administration behavior. **(C)** Chloral hydrate had no significant effect on the weight in rats. **(D)** Chloral hydrate did not change locomotor activity and anxiety mood.

We next examined the potential side effects of CH on the behavior and locomotor ability of rats. Sucrose self-administration test was adopted to evaluate the effect of chloral hydrate on the seeking of natural reward (Figure 5A). The open field experiment was used to evaluate the effect of chloral hydrate on the locomotor activity and anxiety level. The results showed that CH has no effect sucrose seeking and taking [treatment effect, F_1, 13_ = 1.483, *p* = 0.25, Figure 5B], indicating that it does not affect seeking of natural reward. There was no difference in the body weights between the two groups [treatment effect, F_(1, 13)_ = 0.7355, *p* = 0.41, Figure 5C]. The open field test showed that CH did not change the locomotor activity and anxiety level of rats (Figure 5D, central time, *p* = 0.40, moving distance, *p* = 0.80).

## Discussion

In this study, we discovered that CH dose-dependently ameliorated Meth-induced conditioned place preference, as well as Meth self-administration behavior. Single dose (100 mg/kg) treatment of chloral hydrate was able to reduce rat self-administration behavior. Furthermore, CH also effectively prevented cue-induced Meth reinstatement in rats. With 5 consecutive days of CH administrations during the withdrawal period, the rats exhibited reduced drug-seeking and drug-taking behavior in self-administration paradigm. This indicates that chloral hydrate may play an anti-addictive role at the whole stages of Meth self-administration in rats.

Chloral hydrate is a clinically used sedative drug. To discriminate its anti-addiction effect from its sedative effect, we administrated rats with CH 22 h before behavior test. In addition, we examined the effects of chloral hydrate treatment on the natural reward, locomotor activity, and the level of anxiety in rats. The results showed that chloral hydrate treatment only slightly affected the driving force of the rats toward natural reward acquisition, whereas it had less effect on the locomotor activity. In addition, CH didn’t affect the body weights and anxiety levels of the rats, indicating that chloral hydrate treatment is safe in general.

The whole-brain c-fos immunostaining revealed that CH treatment reduced the activities of the claustrum, the dorsal endopiriform nucleus and the rhomboid thalamic nucleus, while enhanced the activity of the zona incerta. The changes in claustrum were the most obvious. Claustrum projects to the PFC and inhibiting the activity of this projection could control the Meth-induced rat’s impulsive behavior (Liu et al., 2019). A significant decrease in the availability of D2/3 receptors in the claustrum of methamphetamine-dependent patients has also been reported in human studies, and genetic studies have shown that genes involved in cocaine and nicotine addiction are specifically expressed in the claustrum (Lee et al., 2009; Liu et al., 2019).

Besides claustrum, the other brain regions with obvious c-fos alterations, such as dorsal endopiriform nucleus and rhomboid thalamic nucleus, also have close relation to drug addiction and dorsal endopiriform nucleus, which has large associations with the function of claustrum, in the brain paralleling information related to the regulation of the limbic system and motor function, both with some functional homogeneity (Watson et al., 2017; Smith et al., 2019). The rhomboid thalamic nucleus undertakes the function of information transmission between mPFC and hippocampus, and spatial memories, working memory, and advanced cognitive ability are related to this brain area (Vertes et al., 2007; Hembrook and Mair, 2011).

Because the activity of the rhomboid thalamic nucleus decreased in the CH treatment group, it is speculated that the rhomboid thalamic nucleus may affect the stability and recurrence of addictive memory. Zona incerta is widely connected to other regions. Its activation and inhibition may be related to the control of stereotyped behavior caused by amphetamines (Supko and Wallace, 1992) and also have the potential to participate in the process of drug addiction. In addition, the traditional addiction-related brain regions, such as the nucleus accumbens and the medial prefrontal cortex, showed a trend of changes without statistical significance. These results suggest that CH exerts its anti-addiction effect might be not through these classical addiction-related brain regions.

In conclusion, 100 mg/kg chloral hydrate significantly reduced methamphetamine self-administration and cue-induced relapse in rats. These effects do not appear to be the result of sedation or dyskinesia. Although the pharmacological mechanism of chloral hydrate is complex and not fully understood, the current results support further testing the potential of these compounds for the treatment of Meth use disorders. Chloral hydrate is a classic drug with a long history of use in the clinic, especially in pediatric testing, The dose we used in the study is the range of clinically permissible dose (Wilson et al., 2014; Mataftsi et al., 2017; Fong et al., 2021), so its safety can be certified and it is possible to conduct clinical testing, to develop the value of new use of its old drugs. In addition, this study further suggests that some sedative drugs previously used in the clinic may be anti-amphetamines with development potential for controlling the harm of synthetic drugs, especially Meth and other psychostimulants. It is of great significance to guide the development of drugs to treat addiction in the future.

## Data Availability Statement

The original contributions presented in this study are included in the article/Supplementary Material, further inquiries can be directed to the corresponding author/s.

## Ethics Statement

The animal study was reviewed and approved by the Institutional Animal Care and Use Committee (IACUC) of Shenzhen Institute of Advanced Technology, Chinese Academy of Sciences.

## Author Contributions

CJ conducted behavior experiments. CJ, YX, JZ, JW, and JH performed histological experiments. CJ, WX, and YZ analyzed the data. CJ, WX, and YZ wrote the manuscript. All authors contributed to the article and approved the submitted version.

## Conflict of Interest

The authors declare that the research was conducted in the absence of any commercial or financial relationships that could be construed as a potential conflict of interest.

## Publisher’s Note

All claims expressed in this article are solely those of the authors and do not necessarily represent those of their affiliated organizations, or those of the publisher, the editors and the reviewers. Any product that may be evaluated in this article, or claim that may be made by its manufacturer, is not guaranteed or endorsed by the publisher.
