# Correction: Chloral hydrate alters brain activation induced by methamphetamine-associated cue and prevents relapse

**DOI:** 10.3389/fnmol.2026.1895561

**Published:** 2026-06-24

**Authors:** Chenyu Jiang, Yunlong Xu, Jiafeng Zhong, Junyan Wu, Jian He, Wei Xu, Yingjie Zhu

**Affiliations:** 1 Shenzhen Key Laboratory of Drug Addiction, Shenzhen Neher Neural Plasticity Laboratory, The Brain Cognition and Brain Disease Institute, Shenzhen Institute of Advanced Technology, Shenzhen-Hong Kong Institute of Brain Science-Shenzhen Fundamental Research Institutions, Chinese Academy of Sciences, Shenzhen, China; 2 College of Life Science, University of Chinese Academy of Sciences, Beijing, China; 3 The First Affiliated Hospital, Sun Yat-sen University, Guangzhou, China; 4 Medical College of Acupuncture-Moxibustion and Rehabilitation, Guangzhou University of Chinese Medicine, Guangzhou, China; 5 Department of Anesthesiology, The First People's Hospital of Foshan, Foshan, China; 6 Faculty of Life and Health Sciences, Shenzhen Institute of Advanced Technology, Chinese Academy of Sciences, Shenzhen, China; 7 CAS Center for Excellence in Brain Science and Intelligence Technology, Chinese Academy of Sciences, Shanghai, China; 8 CAS Key Laboratory of Brain Connectome and Manipulation, Brain Cognition and Brain Disease Institute (BCBDI), Shenzhen Institute of Advanced Technology (SIAT), Chinese Academy of Sciences, Shenzhen, China

**Keywords:** chloral hydrate (CH), methamphetamine, brain activation, cue-induced relapse, addiction

In the published article, in the Section Materials and methods, the source of pentobarbital sodium was not clearly indicated and the composition of the catheter flushing solution was erroneously omitted.

In the Section Materials and methods, subsection *c-fos Immunostaining*, paragraph 1, the sentence “Then, 1 h after the cue-induced reinstatement test, rats were deeply anesthetized by pentobarbital sodium (80 mg/kg, i.p.) and perfused with phosphate buffered saline followed by 4% paraformaldehyde (PFA).” should instead be “Then, 1 h after the cue-induced reinstatement test, rats were deeply anesthetized by pentobarbital sodium (80 mg/kg, i.p., Merck, cat. no. P11011) and perfused with phosphate buffered saline followed by 4% paraformaldehyde (PFA).”

In the Section Materials and methods, subsection *Intravenous Catheterization*, the sentence “Immediately after the surgery, catheters were flushed with 0.2 ml of (2 mg/ml; Sigma) diluted in sterile heparinized saline (10 U/ml; Tocris; Cat. No. 2812/100).” should instead be “Immediately after the surgery, catheters were flushed with 0.2 ml of gentamicin sulfate (2 mg/ml; Sigma; cat. no. E003632) diluted in sterile heparinized saline (10 U/ml; Tocris; cat. no. 2812/100).”

The original version of this article has been updated.

