# Supplementary material for: Chloral Hydrate Alters Brain Activation Induced by Methamphetamine-Associated Cue and Prevents Relapse
Source: Front Mol Neurosci. 2022 Jul 11;15:934167. doi: 10.3389/fnmol.2022.934167 (PMC9309691; doi:10.3389/fnmol.2022.934167)
Supplement: Supplementary file 1 [file Table_1.DOCX]

Table 1

| Statistics for the c-fos expression after relapse test | | | | | | |
| --- | --- | --- | --- | --- | --- | --- |
| Brain region | P Value | Mean  (saline group) | Mean  (CH group) | Difference | SE of difference | t ratio |
| Rh | 0.0055 | 61.67 | 16.67 | -45 | 8.26 | 5.448 |
| dEn | 0.0311 | 129 | 71 | -58 | 17.79 | 3.261 |
| Cl | 0.0363 | 529.3 | 179.7 | -349.7 | 112.9 | 3.098 |
| ZI | 0.0374 | 43.33 | 115.3 | 72 | 23.49 | 3.066 |
| NAc | 0.1321 | 318.7 | 170.7 | -148 | 78.41 | 1.888 |
| mPFC | 0.1628 | 218.3 | 446 | 227.7 | 133.3 | 1.708 |
| LSv | 0.3409 | 133.3 | 68 | -65.33 | 60.49 | 1.08 |
| MS | 0.4257 | 120 | 60 | -60 | 67.73 | 0.8859 |
| DR | 0.513 | 283.3 | 370.3 | 87 | 121.3 | 0.7171 |
| PV | 0.6073 | 94.67 | 114 | 19.33 | 34.71 | 0.5569 |
| VTA | 0.632 | 319.7 | 257.7 | -62 | 119.8 | 0.5176 |
