# Supplementary material for: Chloral Hydrate Alters Brain Activation Induced by Methamphetamine-Associated Cue and Prevents Relapse
Source: Front Mol Neurosci. 2022 Jul 11;15:934167. doi: 10.3389/fnmol.2022.934167 (PMC9309691; doi:10.3389/fnmol.2022.934167)
Supplement: Supplementary file 2 [file Image_1.pdf]

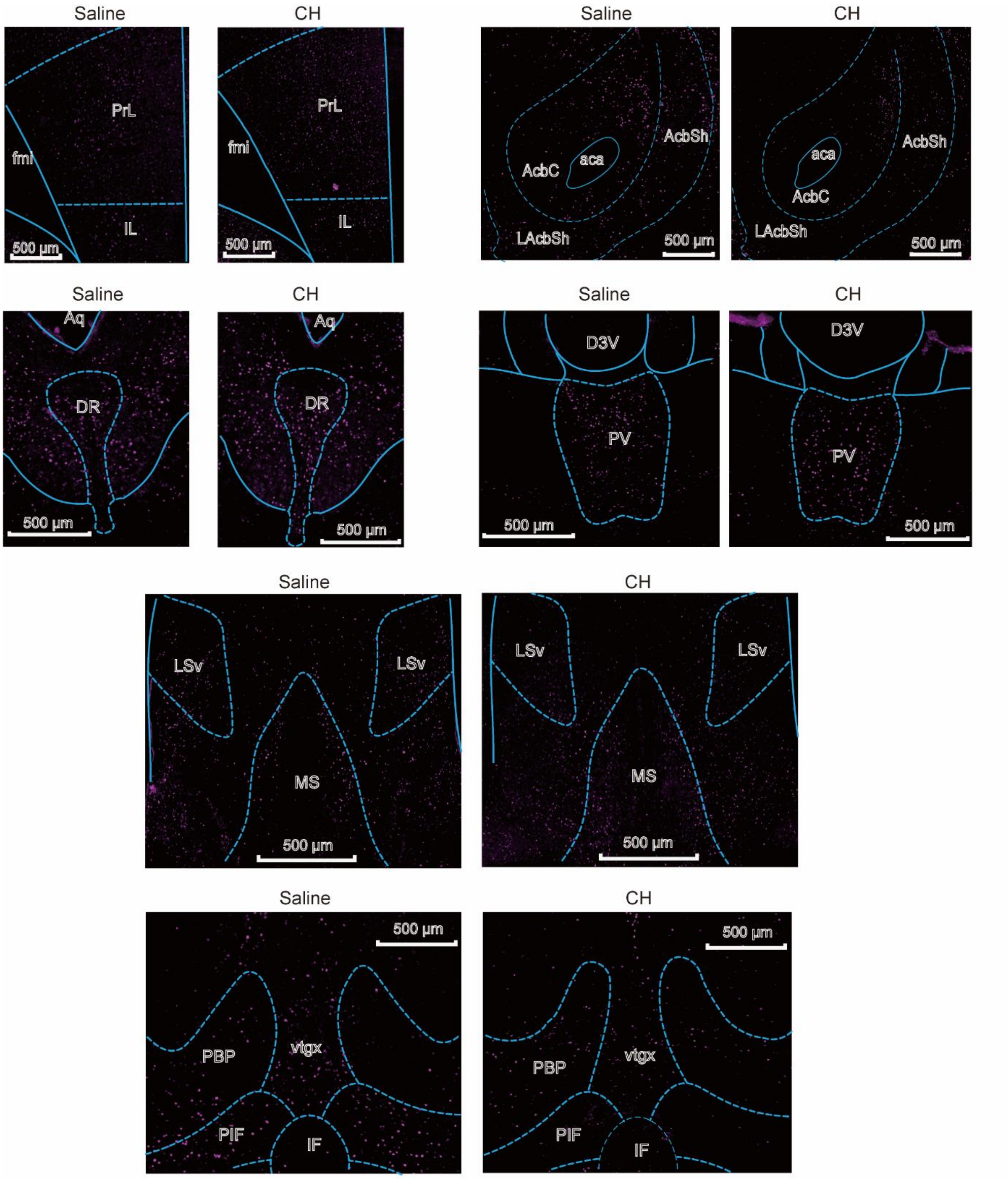

Supplementary figure: The whole-brain c-fos activation after cue-induced Meth relapse in the saline- and CH-treated groups. Abbreviations: prelimbic cortex (PrL), infralimbic cortex (IL), forceps minor of the corpus callosum (fmi), anterior commissure (aca), accumbens nucleus core (AcbC), accumbens nucleus shell (AcbSh), lateral accumbens shell (LAcbSh), aqueduct (Aq), dorsal raphe nucleus (DR), dorsal 3rd ventricle (D3V), paraventricular thalamic nucleus (PV), lateral septal nucleus, ventral part (LSv), medial septal nucleus (MS), parabrachial pigmented nucleus (PBP), parainterfascicular nucleus (PIF), ventral tegmental decussation (vtgx), interfascicular nucleus (IF).
